# Dexmedetomidine as a Protective Agent Against X-Ray Ionizing Radiation-Induced Small Intestinal Injury

**DOI:** 10.3390/antiox14101153

**Published:** 2025-09-23

**Authors:** Süleyman Kalcan, Levent Tumkaya, Tolga Mercantepe, Hamit Yilmaz, Sibel Mataraci Karakas, Ahmet Pergel, Gokhan Demiral, Ali Ozdemir, Sema Rakici

**Affiliations:** 1 Department of General Surgery, Faculty of Medicine, Recep Tayyip Erdogan University, Rize 53100, Turkey; suleyman.kalcan@erdogan.edu.tr (S.K.); ahmet.pergel@erdogan.edu.tr (A.P.); gokhan.demiral@erdogan.edu.tr (G.D.); ali.ozdemir@erdogan.edu.tr (A.O.); 2 Department of Histology and Embryology, Faculty of Medicine, Ondokuz Mayıs University, Samsun 55270, Turkey; levent.tumkaya@erdogan.edu.tr; 3 Department of Histology and Embryology, Faculty of Medicine, Recep Tayyip Erdogan University, Rize 53100, Turkey; tolga.mercantepe@erdogan.edu.tr; 4 Department of Biophysics, Faculty of Medicine, Kahramanmaras Sütcü Imam University, Kahramanmaras 46050, Turkey; 5 Department of Biochemistry, Faculty of Medicine, Recep Tayyip Erdogan University, Rize 53100, Turkey; sibel.karakas@erdogan.edu.tr; 6 Department of Radiation Oncology, Recep Tayyip Erdogan University, Rize 53100, Turkey; sema.rakici@erdogan.edu.tr

**Keywords:** dexmedetomidine, radioprotection, small intestinal injury, oxidative stress, ionizing radiation

## Abstract

Objective: This study was conducted to evaluate the potential radioprotective and therapeutic effects of dexmedetomidine (DEX), a selective α2-adrenergic receptor (α2AR) agonist, against ionizing X-ray-induced small intestinal injury in a dose-dependent manner. Methods: Male Sprague Dawley rats were randomly categorized into four groups. These groups were the Control, Ionizing Radiation (IR, 8 Gy X-ray), IR+DEX 100 µg/kg, and IR+DEX 200 µg/kg. DEX was administered intraperitoneally to the treatment groups 30 min before radiation exposure. All groups were sacrificed 24 h following irradiation. Firstly, the small intestinal tissues were evaluated histopathologically (H&E staining). Subsequently, levels of malondialdehyde (MDA) and glutathione (GSH), as markers of oxidative stress, were measured, and immunohistochemical expression of Caspase-3 and 8-hydroxy-2′-deoxyguanosine (8-OHdG) was analyzed. Results: In the IR group, significant histopathological alterations were observed, including villus atrophy and villus loss due to fusion, crypt loss, and mucosal degeneration. Additionally, there was an increase in MDA levels, a decrease in GSH levels, and a marked elevation in the expression of Caspase-3 and 8-OHdG. In the DEX-treated groups, particularly at the 200 µg/kg dose, significant improvements were noted in these parameters. It was determined that the histological architecture was largely preserved, oxidative stress was reduced, and apoptosis was suppressed. Conclusion: The findings suggest that DEX may effectively reduce X-ray-induced small intestinal injury in a dose-dependent manner, and that this effect is mediated through antioxidant and anti-apoptotic mechanisms. DEX holds potential for the prevention or treatment of radiation-induced gastrointestinal toxicities.

## 1. Introduction

Cancer is among the most significant global health problems in terms of both incidence and mortality rates [1,2]. According to data from the World Health Organization (WHO), approximately 19.3 million new cancer cases were diagnosed in 2020, with nearly 10 million resulting in death. Recent studies indicate that cancer rates are steadily increasing, with projections suggesting a 47% rise by 2040 compared to 2020, reaching an estimated 28.4 million cases [2]. Radiotherapy (RT) is currently one of the most frequently used treatment modalities in cancer management, either as a standalone therapy or in combination with other approaches, depending on the type of cancer and the patient’s condition. It is estimated to be applied in approximately 60% of all cancer cases due to its proven efficacy [3]. Advancements in modern and molecular techniques have enabled the application of RT not only as a monotherapy but also in combination with new-generation treatment modalities, including surgery, antineoplastic therapy, and immunotherapy [4]. Advancements in treatment strategies have significantly improved survival rates. However, despite being a critical modality in cancer therapy, RT is also associated with local and systemic complications that can severely impact patients’ quality of life [5]. Another limitation is the difficulty in determining the dose due to the collateral damage to healthy tissues [6].

The small intestine, which exhibits high radiosensitivity, is the primary dose-limiting organ during abdominal and pelvic RT applications [7]. Radiation enteritis, a condition that arises following irradiation, is a serious clinical problem. Exposure to abdominal or gynecological RT often results in symptoms such as diarrhea, vomiting, abdominal cramps, and weight loss [8]. In patients exposed to 6–8 Gy of IR, gastrointestinal syndrome has been reported, characterized by symptoms including anorexia, fatigue, infection, fluid–electrolyte imbalance, villus atrophy, and gastric retention [9,10]. Studies conducted over the past decade have indicated that radiation-induced damage may occur either through direct DNA strand breaks or indirectly through the increased production of reactive oxygen species (ROS), which has a more destructive effect [5,11]. The increase in ROS leads to a decrease in GSH levels and an increase in MDA, which results in an imbalance of the antioxidant defense system. Moreover, increased ROS also activates the caspase-dependent apoptotic pathway. While the principal advantage of RT is its ability to target tumor tissues, the resulting increase in ROS also causes detrimental effects on the surrounding healthy tissues [6]. Although numerous synthetic and natural radioprotective agents have been developed to mitigate or eliminate these complications, their potential side effects have not been fully prevented. In addition to its use in RT, the protective effects of DEX have been extensively studied in other organs subjected to radiation exposure, including the heart, kidneys, and pancreas [12]. Furthermore, its protective properties on intestinal tissue have been demonstrated in the context of other types of injury, such as ischemia–reperfusion and traumatic brain injury [13], further supporting its potential as a therapeutic agent for a broad range of clinical conditions.

In this study, we aimed to evaluate the radioprotective effect of DEX, a highly selective α2AR agonist, which is currently used in clinical settings due to its antioxidant, anti-inflammatory, anti-apoptotic [14,15], and analgesic properties, and is considered a promising candidate for future multi-organ protection therapies, particularly in the context of small intestinal exposure, which is known for its high radiosensitivity.

## 2. Materials and Methods

Ethical approval for this study was obtained from the Animal Experiments Ethics Committee of Recep Tayyip Erdoğan University (Rize, Türkiye; Approval No: 2023/14, 14 February 2023). 32 male Sprague-Dawley rats (approximately 95 days old, weighing 250–280 g) were obtained from the Experimental Animal Unit of Recep Tayyip Erdoğan University for use in the experiment. The animals were housed under optimal conditions (22 ± 2 °C, 50–55% relative humidity, and a 12 h light/12 h dark photoperiod), with eight rats per cage. All rats were kept under free-moving conditions within the cages and had ad libitum access to food and water [16]. The animals were randomly assigned to four groups, each consisting of eight animals. *Group I (Control):* No treatment was administered. *Group II (IR):* A single dose of 8 Gy whole-body ionizing radiation (IR) was administered. *Group III (Low dose, IR+DEX-1):* 100 µg/kg DEX was administered 30 min prior to radiation, followed by a single dose of 8 Gy whole-body IR. *Group IV (High dose, IR+DEX-2):* 200 µg/kg DEX was administered intraperitoneally 30 min prior to radiation, followed by a single dose of 8 Gy whole-body IR [17].

Drugs & Chemicals

DEX (PRECEDEX 200 µg in a 2 mL vial, Hospira Inc., Clayton, NC, USA) was administered intraperitoneally to rats 30 min prior to irradiation (IR), followed by induction of anesthesia with 50 mg/kg ketamine hydrochloride (Ketalar^®^, Eczacıbaşı Parke-Davis, Istanbul, Turkey) and 10 mg/kg xylazine HCl (Alfazyne^®^, Alfasan International B.V., Woerden, The Netherlands); under anesthesia, the relevant small intestine tissue and blood samples were obtained, and the rats were subsequently euthanized under general anesthesia.

X-ray Irradiation Procedure

The rats were placed in the prone position under anesthesia. Before X-ray irradiation, computed tomography (CT) scans were performed, and conformal treatment planning was performed using the planning system (CMS Xio, version 13.2; Stockholm, Sweden). The rats were subjected to a single-fraction external X-ray irradiation at a dose of 8 Gy using a linear accelerator (Linac) model Elekta Synergy (Elekta, Crawley, UK) operating at 6 MV photon energy. The procedure was performed using an isocentric technique, and the source-to-skin distance (SSD) was set at 100 cm. A 1 cm thick bolus material was placed over each rat, and irradiation was applied from anterior and posterior directions with gantry angles of 0° and 180°, respectively, covering an area of 20 × 20 cm [18].

### 2.1. Biochemical Analysis

#### 2.1.1. Tissue Sampling and Homogenization

A 1 g tissue sample, obtained from a region near the jejunum–ileum junction of the small intestine, was homogenized on ice in 9 mL of 0.15 M KCl solution. The resulting homogenates were homogenized on ice at 9500 rpm for several minutes. Subsequently, the homogenates were centrifuged at 4000× *g* for 10 min at +4 °C, and the supernatants were collected’ [18]. MDA and GSH levels of the supernatants were then determined spectrophotometrically.

#### 2.1.2. Malondialdehyde Analysis

MDA levels were determined using the method of Ohkawa et al. [19]. MDA, a byproduct of lipid peroxidation, was determined by the formation of a pink chromogen in Tris-buffered saline solution upon heating, and the absorbance was read spectrophotometrically at 532 nm [1].

#### 2.1.3. Glutathione Analysis

GSH levels in the small intestinal tissue were determined using Ellman’s reagent method [20]. A total of 200 µL of 3M Na_2_HPO_4_ and 50 µL of DTNB (prepared by dissolving 4 mg of DTNB in 10 mL of 1% sodium citrate) were added to 50 µL of supernatant. The resulting mixture was vortexed and the absorbance was measured at 412 nm using a spectrophotometer.

### 2.2. Histopathological Analysis

Tissue specimens obtained from the region proximal to the jejunum–ileum junction of the small intestine were carefully trimmed and oriented before histological processing, and subsequently fixed in 10% neutral buffered formalin solution for 24 h. Following fixation, the tissues were dehydrated through a graded ethanol series (Merck GmbH, Darmstadt, Germany) using an automated tissue processor (Shandon Citadel 2000, Thermo Scientific, Darmstadt, Germany).

In the subsequent step, the samples were cleared in two consecutive xylene baths (Merck GmbH, Germany) and subjected to an infiltration protocol involving both soft and hard paraffin (Merck GmbH, Germany). After paraffin infiltration, the tissues were held overnight in molten paraffin and then embedded in tissue cassettes (Isolab GmbH, Eschau, Germany) using an embedding unit (Leica Biosystems EG1160, Nussloch, Germany).

Tissue samples were obtained from the paraffin blocks using a rotary microtome (RM2525, Leica Biosystems, Germany) at a thickness of 2–3 µm. The sections were stained using a fully automated stainer (Leica ST5010, Leica Biosystems, Nussloch, Germany) with Harris hematoxylin (Merck GmbH, Germany) and Eosin G (Merck GmbH, Germany), and prepared for microscopic evaluation.

### 2.3. Immunohistochemical (IHC) Staining Procedure

In this study, Caspase-3 (rabbit polyclonal, ab11419, Abcam, Cambridge, UK) was used to identify apoptotic cells, and 8-OHdG primary antibody (rabbit polyclonal, ab48508, Abcam, UK) was used to detect oxidative stress. Secondary antibody kits were also used in conjunction with the primary antibodies.

To eliminate endogenous peroxidase activity in the sections, incubation was performed with 3% hydrogen peroxide (H_2_O_2_) solution for 15 min. This step was carried out using a fully automated Bond MAX IHC/ISH system (Leica Biosystems, Sydney, NSW, Australia).

Subsequently, the tissue sections were treated with a secondary blocking solution for 20 min to prevent non-specific staining. Following this, the sections were incubated with the primary antibody at room temperature for 60 min.

After primary antibody application, HRP-conjugated secondary antibodies were applied, and the chromogenic reaction was initiated by adding DAB (3,3′-diaminobenzidine) solution (Abcam, UK). Color development was monitored under a light microscope.

Following staining, the tissue sections were counterstained with Harris hematoxylin (Merck, Germany) and then mounted using appropriate media and coverslips. The prepared slides were archived for evaluation under a light microscope.

### 2.4. Semi-Quantitative Evaluation

Small intestinal tissue sections stained with hematoxylin and eosin were examined under a light microscope. Based on findings such as villus fusion, villus shortening, inflammation, and hemorrhage, the intestinal histopathological damage score (IHDS) was calculated in accordance with previous studies on intestinal tissue injury (Table 1) [18,21]. Enterocytes and goblet cells showing positivity for Caspase-3 and 8-OHdG were analyzed by two blinded histopathologists (T.M., L.T.) as shown in Table 2. For each rat, 30 different fields were randomly selected from the slide, and the IHC positivity score was measured.

### 2.5. Statistical Analysis

Statistical analyses were performed using SPSS Software v 22.0 for Windows (IBM Corp., Armonk, NY, USA). Data normality was verified using the Shapiro–Wilk test, and descriptive values were expressed as mean ± SD. Homogeneity of variances across groups was tested using Levene’s test. When variances were homogeneous, one-way ANOVA (Tukey’s HSD post hoc test) was applied. In cases of non-homogeneous variances, Welch’s test (Tamhane’s T2 post hoc test) was used. Scores were reported as median (min–max). Group score comparisons were performed using the Kruskal–Wallis test. Statistical significance was considered at *p* < 0.05 for all analyses.

## 3. Results

### 3.1. Biochemical Analysis

In the control group, the MDA level was determined as 13.46 ± 0.99 nmol/mg tissue, whereas it increased to 17.04 ± 2.57 nmol/mg tissue in the IR group (*p* = 0.03; Table 3). The MDA levels in the IR+DEX 100 and IR+DEX 200 groups were found to decrease to 8.96 ± 2.30 and 7.66 ± 1.51 nmol/mg tissue, respectively. This reduction was statistically significant when both treatment groups were compared to the control group (*p* = 0.003; *p* < 0.001; Table 3). Similarly, when compared to the IR group, MDA levels in the IR+DEX 100 and IR+DEX 200 groups also showed a statistically significant decrease (*p* = 0.000; *p* < 0.001; Table 3).

In the control group, the GSH level was measured as 11.54 ± 2.45 nmol/mg tissue, while in the IR group, it decreased to 6.45 ± 1.04 nmol/mg tissue (*p* = 0.02; Table 3). When the control group was compared to the IR+DEX 100 and IR+DEX 200 groups, the GSH levels were found to be 8.31 ± 2.02 and 9.86 ± 1.70 nmol/mg tissue, respectively. A comparison between the IR+DEX groups and the IR group revealed an increase in GSH levels; however, a statistically significant difference was observed only between the IR+DEX 200 group and the IR group (*p* = 0.03; Table 3).

### 3.2. Histopathological Analysis

**Figure 1:**
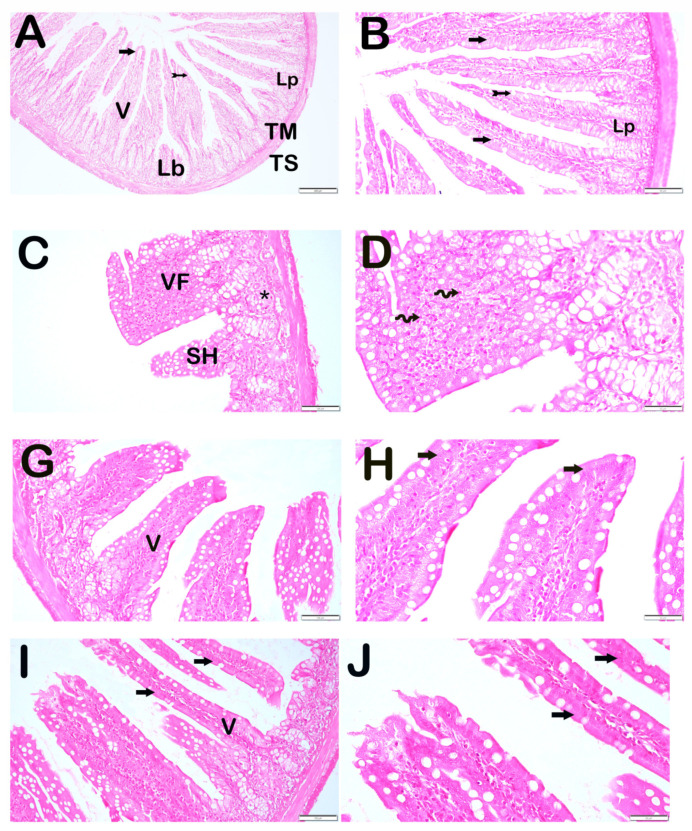
Representative light microscopic images of ileal tissue sections stained with Harris hematoxylin and Eosin G. Villus (V), Lamina propria (LP), Lieberkühn Crypts (LB), Tunica muscularis (TM), Tunica Serosa (TS). **Control group** (**A**) **(×10)**, (**B**) **(×20):** Long villi structures containing normal enterocytes (arrow) and goblet cells (tailed arrow) were observed (Intestinal Histopathological Damage Score *[IHDS]: 0 [0–1]*). **IR group** (**C**) **(×20)**, (**D**) **(×40):** Shortened villi (SH) and fused villi (VF) were observed. Inflammatory infiltration and edematous areas (asterisk) were noted in the lamina propria (*IHDS: 7.5 [6–8]*). **IR+DEX 100 group** (**G**) **(×20)**, (**H**) **(×40):** A reduction in villus shortening, villus fusion, inflammation, and edema was observed (*IHDS: 2 [2–3]*). **IR+DEX 200 group** (**I**) **(×20)**, (**J**) **(×40):** Villi containing typical enterocytes and goblet cells were observed in the tunica mucosa. A reduction in villus fusion, inflammation, and edematous areas was noted (*IHDS: 2 [2–3]*).

Upon light microscopic examination of ileum tissue sections stained with Harris hematoxylin and Eosin G, villus structures containing normally structured enterocytes and goblet cells were observed in the tunica mucosa of the control group (Figure 1A,B, Table 4, IHDS: 0 [0–1]).

In contrast, in the intestinal tissue sections of the X-ray irradiation group, a reduction in the number of villi and shortening of villi due to villus fusion were detected. In addition, widespread infiltrative areas and edematous regions were observed in the lamina propria (Figure 1C,D, Table 4, *p* = 0.001, IHDS: 7.5 [6–8]).

On the other hand, in the IR+DEX 100 group, reductions in villus shortening, villus fusion, inflammation, and edematous areas were observed in the tunica mucosa of the intestinal tissue (Figure 1G,H, Table 4, *p* = 0.001, IHDS: 2 [2–3]). In the IR+DEX 200 group, villi containing typical enterocytes and goblet cells were widely observed in the tunica mucosa. Additionally, decreases in villus fusion, inflammation, and edematous areas were also noted (Figure 1I,J, Table 4, *p* = 0.001, IHDS: 2 [2–3]).

### 3.3. IHC Analysis

#### 3.3.1. Caspase-3 Positivity

**Figure 2:**
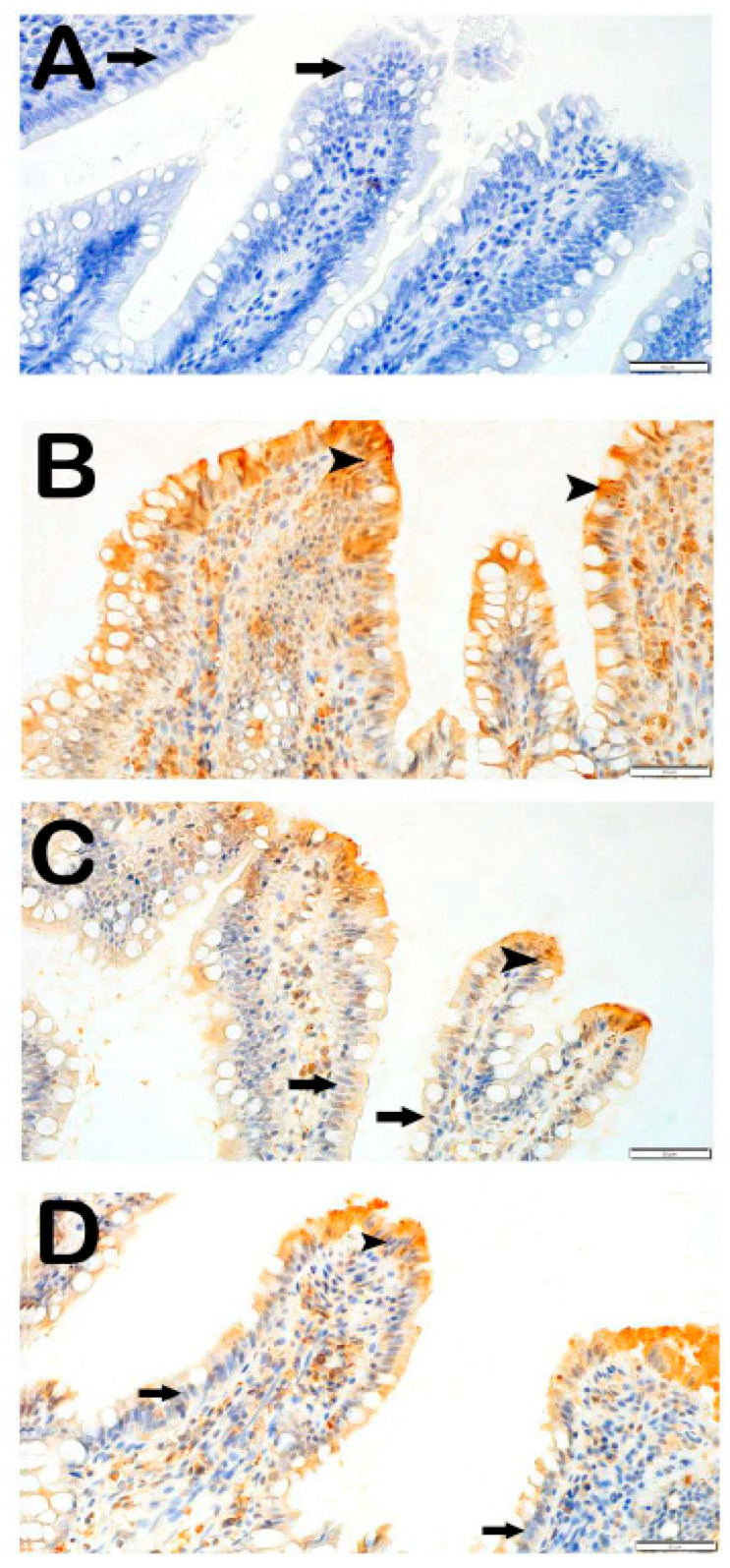
Representative light microscopic images of intestinal tissue sections incubated with Caspase-3 primary antibody. (**A**) **(×40): Control group:** Caspase-3 immunonegativity is observed in the enterocytes with normal morphology in the tunica mucosa **(arrow)** *(Caspase-3 positivity score: 0 [0–1])*. (**B**) **(×40): IR group:** Apoptotic enterocytes showing strong Caspase-3 immunopositivity are visible in the villi **(arrowhead)** *(Caspase-3: 3 [2–3])*. (**C**) **(×40) IR+DEX 100 group:** A reduced number of Caspase-3 immunopositive enterocytes are observed in the villi compared to the IR group **(arrowhead)** *(Caspase-3 positivity score: 1 [1–1])*. (**D**) **(×40) IR+DEX 200 group:** Apoptotic enterocytes in the tunica mucosa showing Caspase-3 immunopositivity are markedly decreased **(arrow)** *(Caspase-3 positivity score: 1 [0–1])*.

In the intestinal tissue sections of the control group incubated with the Caspase-3 primary antibody, enterocytes and goblet cells were observed to be immunonegative for Caspase-3 (Figure 2A, Table 5, Caspase-3 positivity score: 0 [0–1]).

In contrast, in the IR group, compared to the control group, an increased number of apoptotic enterocytes and apoptotic goblet cells showing strong immunopositivity for Caspase-3 were observed (Figure 2B, Table 5, *p* = 0.001, Caspase-3: 3 [2–3]).

In the treatment groups, the IR+DEX 100 group showed a reduction in the number of enterocytes with strong Caspase-3 immunopositivity in the villi compared to the IR group (Figure 2C, Table 5, *p* = 0.006, Caspase-3: 1 [1–1]). In the IR+DEX 200 group, a decrease in the number of apoptotic enterocytes and goblet cells with strong Caspase-3 immunopositivity was observed in the tunica mucosa of intestinal tissue sections compared to the IR group (Figure 2D, Table 5, *p* = 0.001, Caspase-3: 1 [0–1]).

#### 3.3.2. 8-OHdG Positivity

**Figure 3:**
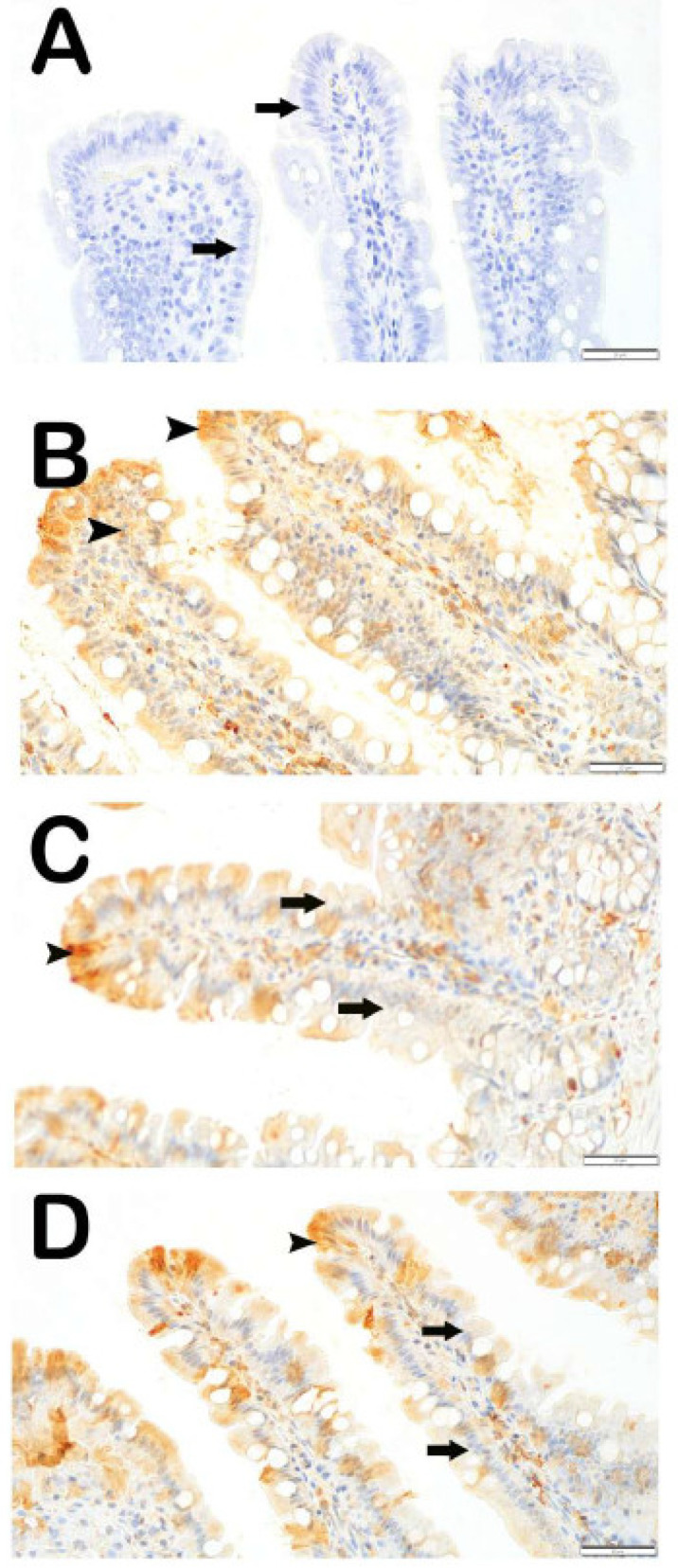
Representative light microscopic images of intestinal tissue sections incubated with 8-OHdG primary antibody. (**A**) **(×40): Control group:** Enterocytes in the villi of intestinal tissue sections exhibit immunonegativity for 8-OHdG **(arrow)** *(8-OHdG positivity score: 0 [0–0])*. (**B**) **(×40): IR group:** A high number of enterocytes showing strong immunopositivity for 8-OHdG are observed in the villi **(arrowhead)** *(8-OHdG: 2 [2–3])*. (**C**) **(×40) IR+DEX 100 group:** A reduced number of enterocytes immunopositive for 8-OHdG are observed in the villi **(arrowhead)** *(8-OHdG: 0 [0–1])*. (**D**) **(×40) IR+ DEX 200 group:** A marked reduction in the number of enterocytes immunopositive for 8-OHdG is observed in intestinal tissue sections **(arrow)** *(8-OHdG: 0 [0–0])*.

In the light microscopic examination of small intestinal tissues incubated with 8-OHdG, sections from the control group showed that enterocytes in the villi had a normal structure and were immunonegative for the 8-OHdG primary antibody (arrow) (Figure 3A, Table 5, 8-OHdG positivity score: 0 [0–0]). In contrast, in the X-ray irradiation group, the number of enterocytes and goblet cells showing strong immunopositivity for the 8-OHdG primary antibody was markedly increased compared to the control group (Figure 3B, Table 5, *p* = 0.001; 8-OHdG: 2 [2–3]).

In the treatment groups, when the IR+DEX 100 group was compared to the IR group, a reduction was observed in the number of enterocytes and goblet cells showing strong 8-OHdG immunopositivity in the villi (Figure 3C, Table 5, *p* = 0.001; 8-OHdG: 0 [0–1]). Similarly, in the IR+DEX 200 group, the number of enterocytes with strong 8-OHdG immunopositivity in intestinal tissue sections was found to be reduced compared to the IR group (Figure 3D, Table 5, *p* = 0.001; 8-OHdG: 0 [0–0]).

## 4. Discussion

In RT applications, the small intestine, being a highly radiosensitive and dose-limiting organ, can be damaged due to exposure to IR. Intestinal barriers play a critical role in protecting the body against pathogenic conditions such as toxins, inflammation, stress, and surgical trauma. Intestinal injury can facilitate the translocation of bacteria and toxins to mesenteric lymphoid tissues, lymphatic fluid, the bloodstream, and distant organs, potentially triggering gut-derived sepsis and subsequent multiple organ dysfunction syndrome (MODS) [22]. Taking into account the dose-limiting nature of the intestine during IR application may help reduce the incidence of sepsis, improve clinical prognosis, and decrease mortality rates in critically ill patients. El-Ghazaly et al. reported that exposure to 6 Gy IR caused villus fusion due to intestinal mucositis and epithelial cell shedding [23]. El-Ghazaly et al. [23] reported that exposure to 6 Gy IR caused villus fusion due to intestinal mucositis and epithelial cell shedding.

These considerations inevitably raise a critical question in the context of RT: To what extent can DEX, when administered systemically as a radioprotective agent, selectively protect healthy tissues without diminishing the therapeutic efficacy against tumor cells? Addressing this issue is essential to understanding the translational potential of DEX in clinical oncology.

The role of DEX in this context is attributed to its multifaceted mechanisms of action. Notably, the differential expression of α2AR between tumor and normal tissues [24,25] highlights the agent’s potential to confer a more pronounced protective effect on healthy tissues. Furthermore, given that tumor hypoxia is a well-established driver of radioresistance, it has been postulated that the vasodilatory properties of DEX may improve perfusion and oxygenation within the tumor microenvironment, thereby sensitizing malignant cells to radiation therapy [26]. Of particular importance, emerging evidence from recent in vitro and preclinical investigations indicates that DEX may also exert direct antitumor effects by promoting immunogenic cell death and enhancing tumor-specific immune responses [27]. Collectively, these findings suggest that the clinical use of DEX may offer radioprotective advantages while not only preserving but potentially augmenting its therapeutic efficacy against tumors.

In the present study, DEX was administered at 100 and 200 µg/kg based on previous preclinical investigations demonstrating its pharmacological and protective effects in rats [17,28,29]. While these doses are higher than those typically approved for clinical use in humans, they were selected to achieve the desired protective effect in the animal model. Current clinical guidelines indicate that the maximum recommended intravenous dose in humans corresponds to roughly half of the highest rat dose when adjusted for body surface area [30]. Thus, the doses applied in this study are appropriate for preclinical evaluation. Nevertheless, direct extrapolation to human applications is not possible, and further studies are warranted to assess safety and efficacy in clinical settings.

However, compared to clinically approved and proven radioprotective agents, dexmedetomidine is not yet accepted as a standard treatment option. For example, amifostine reduces radiation-induced side effects in head and neck cancer treatment [31], while *N*-acetylcysteine (NAC) protects cardiac and renal tissues from oxidative damage [32].

The radioprotective effects of dexmedetomidine have been demonstrated in preclinical studies [33] and have mostly been administered at doses above clinical levels. Therefore, more clinical studies are needed for its use as a safe and effective radioprotective agent.

In light of the current literature, although dexmedetomidine shows promising results at the preclinical level, its efficacy and safety in clinical practice have not yet been sufficiently proven; the efficacy of other agents at clinical doses is supported, and comparative studies are needed before the clinical use of dexmedetomidine.

This study demonstrates that pretreatment with DEX may provide a radioprotective effect against small intestinal injury, which is highly susceptible to both acute and chronic toxicity induced by IR due to its rapid epithelial turnover. Our findings are consistent with previous studies reporting the anti-inflammatory, anti-apoptotic, and antioxidant effects of DEX in the small intestine [34,35]. These effects of DEX also play an active role in intestinal protection. HE, Guo-Zun et al. reported that both pretreatment and increasing doses of DEX were more protective in their evaluation of the efficacy of DEX against small intestinal injury at different doses and durations [36]. In the same study, in addition to a reduction in oxidative stress, levels of MDA and myeloperoxidase (MPO) as inflammatory markers, serum levels of diamine oxidase (DAO), caspase-3 activity, ileal mucosal thickness, and the apoptotic in DEX of ileal mucosal cells were found to be significantly lower in the DEX-treated group compared to the untreated group. Although the mechanism of IR-induced acute gastrointestinal syndrome has not been fully elucidated, recent studies suggest that increased oxidative stress due to elevated ROS levels may be a contributing factor. Furthermore, numerous studies have declared that increased ROS production plays a critical role in the detrimental effects of IR by leading to elevated MDA levels and reduced GSH levels [10,24].

Although further investigation is needed to elucidate the exact mechanism underlying the efficacy of DEX administration before or after IR, the histopathological findings in our study indicate that structural damage induced by IR, such as villus loss, fusion, and shortening, was significantly attenuated. In particular, the integrity of enterocytes was preserved, and the inflammatory response was alleviated at the 200 µg/kg dose. These results highlight the protective effect of DEX on the gastrointestinal mucosa. Moreover, Caspase-3 expression, indicative of radiation-induced apoptosis, and 8-OHdG positivity, a marker of oxidative DNA damage, were significantly reduced with DEX treatment. These findings suggest that DEX mitigates IR-induced intestinal injury through anti-apoptotic and antioxidant mechanisms. Additionally, compared to the control group, GSH levels were higher and MDA levels were lower in DEX-treated groups, with a significantly greater antioxidant capacity observed in the 200 µg/kg group compared to the 100 µg/kg group. Our findings are consistent with recent studies reporting the intestinal protective effects of DEX [34].

Several studies have shown that the effects of DEX on oxidative stress can be modulated via α_2_-adrenoceptors. Weng and colleagues (2018) [37] reported that α_2_-adrenoceptor activation has antioxidant effects. Similarly, Zhang et al. (2022) [38] reported that DEX reduced oxidative stress and inflammation in vascular smooth muscle cells via the α_2_-adrenoceptor/GSK-3β/MKP-1/NRF2 pathways. Lv and colleagues (2019) [39] also demonstrated that DEX improves acute intestinal injury by activating the Nrf2/HO-1 antioxidant pathways via α_2_-adrenoceptors. These studies establish a consistent literature basis for the potential mechanism of DEX’s antioxidant effects.

In the current study, however, it was not directly tested whether these effects of DEX occur specifically via α_2_-adrenoceptors; our study was designed on an acute model, and the opportunity to directly examine the mechanistic details was limited. Nevertheless, our findings support the potential effects of DEX on oxidative stress and indicate that the mechanism of this effect should be investigated in further studies. In this context, the current literature and our findings together provide a consistent and reinforced framework regarding the possible mechanism of the antioxidant effects of DEX.

In addition to promoting intestinal motility and improving microcirculation, DEX also protects the intestinal epithelial barrier against damage [15]. This beneficial effect is mediated by the enhancement of intestinal smooth muscle contractions. DEX may reduce enterogastric peristalsis under normal conditions via the enteric neurons [40]. However, in pathological states like infection, stress, or trauma, DEX supports intestinal microcirculatory perfusion, which in turn helps preserve the intestinal barrier and restore gastrointestinal motility [41]. This effect is achieved through the reduction in inflammation and stress responses, maintenance of hemodynamic stability, alleviating postoperative pain, and lowering postoperative opioid requirements [15]. Recent animal studies have reported that DEX increases the number of perfused capillaries in the intestinal mucosa and muscle layers. As a result, it helps prevent disruption of the epithelial barrier and alleviates microcirculatory disturbances [42,43].

## 5. Conclusions

This pilot study demonstrates the radioprotective potential of DEX, indicating its ability to attenuate radiation-induced gastrointestinal complications such as diarrhea, vomiting, abdominal cramps, weight loss, anorexia, fatigue, infection, and fluid–electrolyte imbalances. By modulating oxidative stress and inflammatory pathways, DEX emerges as a promising candidate for gastrointestinal protection, although further studies are necessary to establish comprehensive therapeutic protocols and clarify its clinical relevance.

Several limitations should be acknowledged when interpreting these findings. The use of an acute experimental model restricts conclusions about long-term efficacy, and testing only two doses prevents a full characterization of the dose–response relationship. Moreover, the evaluation of oxidative stress using only two parameters provides a limited view of the complex biological mechanisms involved. While these constraints limit the generalizability of the results, the study offers an important preliminary step. Future investigations using chronic models, expanded dose ranges, and a broader spectrum of oxidative and antioxidative markers are warranted to validate and extend the therapeutic promise of DEX.

## Figures and Tables

**Table 1:** Intestinal Histopathological Damage Score Modified (IHDS).

Score	Findings
Villous Fusion	
0	≤5%
1	≤25%
2	≤50%
3	≤75%
Villous Shortening	
0	≤5%
1	≤25%
2	≤50%
3	≤75%
Inflammation	
0	≤5%
1	≤25%
2	≤50%
3	≤75%
Hemorrhage	
0	≤5%
1	≤25%
2	≤50%
3	≤75%

**Table 2:** Immunohistochemical Positivity Score.

Score	
0	None (less than 5%)
1	Mild (less than 25%)
2	Moderate (less than 50%)
3	Severe (less than 75%)

**Table 3:** Biochemical analysis results (mean ± standard deviation).

Group	MDA (nmol/g Tissue)	GSH (nmol/g Tissue)
Control	13.46 ± 0.99	11.54 ± 2.45
IR	17.04 ± 2.57 **^c^**	6.45 ± 1.04 **^a^**
IR+DEX 100	8.96 ± 2.30 **^d,f^**	8.31 ± 2.02
IR+DEX 200	7.66 ± 1.51 **^e,g^**	9.86 ± 1.70 **^b^**

**^a^** *p* = 0.02; **^c^** *p* = 0.03; **^d^** *p* = 0.003, **^e^** *p* < 0.001: Compared to the Control group. **^b^** *p* = 0.03; **^f^** *p* < 0.001; **^g^** *p* < 0.001: Compared to the IR group One-Way ANOVA: Welch, Tamhane’St2 IR: Ionizing Radiation; DEX: Dexmedetomidine; MDA: Malondialdehyde; GSH: Glutathione.

**Table 4:** IHDS Results (Meidan-25–75% interquartile range).

Groups	Villus Fusion	Shortening of the Villi	Inflammation	Hemorrhage	IHDS
Control	0 (0–0)	0 (0–0)	0 (0–0)	0 (0–0)	0 (0–1)
IR	2.5 (2–3) **^a^**	2.5 (2–3)	1 (1–1) **^a^**	1 (1–1) **^a^**	7.5 (6–8) **^a^**
IR+DEX 100	1 (1–1) **^b^**	1 (0–1) **^b^**	0 (0–0) **^b^**	0 (0–0) **^b^**	2 (2–3) **^a,b^**
IR+DEX 200	1 (1–1) **^b^**	1 (1–1) **^b^**	0 (0–0) **^b^**	0 (0–0) **^b^**	2 (2–3) **^a,b^**

**^a^** *p* = 0.001 Compored to Control group; **^b^** *p* = 0.001 Compored to X-ray Irradittion group Kuskall Wallis/Tamhne T2 test; IHDS: Intestinal Histopathological Damage Score.

**Table 5:** Immuno-histocemical Analysis Results (median-interquartile range 25–75%).

Group	Caspase-3 Positivity Score	8-OHdG Positivity Score
Control	0 (0–1)	0 (0–0)
IR	3 (2–3) **^a^**	2 (2–3) **^a^**
IR+DEX 100	1 (1–1) **^b^**	0 (0–1) **^c^**
IR+DEX 200	1 (0–1) **^c^**	0 (0–0) **^c^**

**^a^** *p* = 0.001 Compored to Control group; **^b^** *p* = 0.006 Compored to Irradittion group; **^c^** *p* = 0.001 Compored to Irradittion group Kuskall Wallis/Tamhne T2 test.

## Data Availability

The datasets used and/or analyzed during the current study are available from the corresponding author upon reasonable request.
